# Dr. Wigan on the Duality of the Mind

**Published:** 1848-04-01

**Authors:** 


ART. II.—*A New View of Insanity. The Duality of the Mind, &c., &c. With an Appendix.* By A. L. WIGAN, M.D. London, Longman and Co., 1844. 8vo, pp. 460. And a Pamphlet (printed for private circulation) entitled, *A Few more Words on the Duality of the Mind, and some of its Corollaries.* 1847.

THE subject we have selected for review is one of that recondite nature which usually puzzles or repulses readers unaccustomed to deep research. For, in its elemental or uneducated condition, the mind finds pleasure only in simple ideas or sounds. That which is composite and elaborate is chosen last of all, because it is the last to be understood and appreciated. The meagre notes of a Scotch jig are of this primitive character, arguing a taste as yet in its low or infant state. The sustained note of the plain chant comes next in order of preference; and difficult as it is of correct and efficient execution, pleases the untutored listener the more because the mind can dwell on each sound as it is uttered, and weigh, or fancy it can weigh, the value and meaning of every note. A higher school succeeds to this, and it is one which, simple as it may seem to be, is, in point of scientific skill and accomplishment, of the highest quality,—we mean, that of the composers from Palestrina to Baini. But how mature, intellectual, and refined is that taste which delights in the subacid chords of a Weber, the melodious grandeur of a Mozart, and the sublime pathos of a Beethoven!

To raise the eyes to heaven on a starlight night, and gaze on the countless host of brilliant points of fire that stud the dark blue, deep infinitude, was once the occupation of the shepherds of old, who named the constellations as they rose and set in their courses from east to west. The astrologer gazed, and calculated, and cast his nativity by the rising and setting of the same celestial wonders: the Dog-star was then, as it is now, the prophet of the scalding heats of summer, and red Orion announced then, as he still continues to do, the weltering storms of autumn, while mounting with disastrous omen to the shrouded heavens. But the modern astronomer is far more highly gifted than the visionary astrologer in his luckiest reveries, or than the silent shepherd at his midnight watch. To measure the masses of the planets as they roll around the sun in their several orbits, and by means of their once accurately determined parallax, to count the number of the days of their years; to predict the minutes of an eclipse, to ascertain the occultation of one of the satellites of Jupiter, and to enumerate the periods of the erratic times of a comet; to supply the mariner with a practical index for exactly reckoning his longitude by the altitude of the sun, as he sails along the pathless ocean, or to form a table of tides according to the moon's age, in language as familiar as household words, is an achievement of science only not deemed marvellous because vernacular, and not divine, because we fancy ourselves superior to the pagans, by refusing to venerate the real benefactors of the human race.

But far more transcendant than all these glories is the mind of man, —encased within its bony tabernacle for a brief and hurried season,—confined to this small spot of earth,—and from beneath the pent-house of its eyelid, peering forth on the broad daylight of this fair world, and glancing with almost angel's ken from heaven to earth, and from earth to heaven. Mind is indeed an enigma, the solution of which is apparently beyond the reach of this very mind, itself the problem, the demonstrator, the demonstration, and the demonstrand. The mental operation is introverted,—the eye must view itself,—the thought must think on thought,—and the mind must understand and explain the mind. O wondrous work! who shall penetrate its inmost recesses, and visit the varied chambers of its imagery? What tongue shall tell the legends of its lore? or what pen describe the mazes of its endless labyrinth of ideas? Pass on, thou slow fleet-footed herald, Time! and guide us to that golden mansion (*domus aurea*) where the hidden things of earth shall be refulgent with truth, and the failing things of age glow with the splendours of an everlasting knowledge!

Impressed with the proper dignity of our subject, we enter upon it, not with diffidence, but with caution, intending to confine ourselves entirely to the facts of the case, and restrict ourselves rigidly to those conclusions justly deducible from irrefragable reasoning. If, in the course of our inquiry, we declare ourselves rather freely, it must not be supposed that we have any desire to contradict an author who deserves *in limine* so much of our gratitude for his efforts, while the public will at the same time grant us the indulgence of believing that our object is not to question established truths, but to investigate and consider a new and difficult question, which we shall leave as we have found it—an open one.

Ὁ *κεφαλος του ανθρωπου εστι διπλος*, the brain of man is double, says Hippocrates, just as it is in all other animals.* In man, certainly, it is a double organ, or, more properly speaking, a *dual;* and, as we shall presently undertake to show, it seems requisite that the brain should be thus a twofold organ for the perfect manifestation of the mind. For in the lowest scale of organic existences, in which the functions of life are scarcely raised above those of vegetation, the cephalic ganglion is only a solitary speck or a single thread. In what manner the mind, as mind, is connected with, or dependent upon, the matter of the brain, it were hard to tell, but that it is thus dependent or connected no one can doubt. Instances are on record† in which the brain has, while in full operation, been so pressed upon as to extinguish the mind for the time being, but in which, as soon as the pressure or disturbing force has been removed, the mind has recovered its elasticity, and resumed its operations at the very point at which they had become suspended. It is

* *De morbo sacro*, vi. 1. This remark of Hippocrates shows that he knew something more of *human* anatomy than what he might have picked up by chance from the dissection of the lower animals; which is still more strongly shown, when, in the next sentence, he declares that it is thus double, *ωσπερ*, *quapropter*, because it is divided in the middle by a thin membrane, *μηνιγξ λεπτη*:—*μηνιγξ* is literally the *pia mater;* but as the *pia mater* does not divide the brain into two halves *per se*, it must mean the *falx major*, which does so divide it. The passage, however, explains itself. In his treatise *De fracturis*, he mentions the clavicle, its sternal and acromial attachments, in terms which none but a *human* anatomist could properly employ.

† Müller's Elements of Physiology, by Baly, 2nd edit., London, 1840, p. 815.

evident that, in such cases, the mind depends upon a certain condition of the particles of the brain.

The lens of the telescope does not constitute in itself the laws of optics, any more than the retina is itself the sight. Nor is the sensation or image conveyed along the optic nerve the sight, any more than the sight can be said to be the final particle of the ultimate molecule of that part of the brain to which the image or picture is at last conveyed. For the power of vision is something beyond the remotest subdivision of the most distant molecule of the neurine, just as the well-known laws of optics are above and beyond the lens, or the equally well-known laws of acoustics are beyond the speaking tube, the trumpet, or the harmonious chords of the harp. But, nevertheless, the mind requires a particular molecule of neurine arranged in a certain mode, and an optic nerve and a retina, and a vitreous humour, and a crystalline lens, and an aqueous fluid, and a transparent cornea, before the power of vision can be rightly expressed or properly conceived within the sensorium. This is not materiality; or else it is materiality and immateriality conjoined; the imponderable principle energizing through the ponderable organ, and the material organ subserving the immaterial essence or *vis vitæ.* The abrupt space that intervenes between the last portion of matter and the earliest dawn of intellect, is an unfathomable gulf tantamount to infinitude—for infinite it must be, since nothing can fill it up. No correct reasoning can conduct us farther than this.

Nor can the mind itself be said to be double, or dual, in the same sense as its organ the brain is said to be, inasmuch as the mind is, like light, electricity, magnetism, &c., a first principle or entity, indivisible and indiscerptible. It cannot be divided, duplicated, multiplied, expanded, or condensed. It is in its own nature one—ων, ens, sum, being, essence. In the monogamic molluscæ, which propagate by spontaneous off-sets or shoots, and not by reciprocal generation, the new creature is not the product of two, but the self-division of one; and yet in these animals, or animalculæ, the life thus apparently multiplied in each creature is, nevertheless, but one and the same life in principle as that from which it sprung. Its being is one, while its mechanism is alone multiplied. Its immaterial being is not manifold, although enunciated by a material organism which is manifold.

In the same manner, the mind may pass from an active to a latent state, and from a latent to an active—as in sleep, coma, suspended animation, seminal fructification, and oval germination, in each of which instances the mind is as positively latent as the electricity in the thunder cloud before it is bolted forth by fulmination, or the fire in the flint before it is struck out into sparks by collision with steel. Now, none of these things can be predicated of the material organ of mind—the brain, which obviously rests upon grounds of inquiry altogether different from the element of which it is only the instrument.

So that the mind may be either active or latent; but not so the brain. The mind cannot be dual, although the brain may. The mind is not the medium of thought, although the matter of the brain is. The mind is the generative principle, indigent of nothing—the brain is the subserving vessel, indigent of everything. The particles of the brain, like those of the body generally, are heterogeneous, dividual, personal, and transient —the mind, on the contrary, is itself a monad, homogeneous, individual, identical, and perpetual.* This is the difference which it was necessary for us to define accurately before entering on our proposed inquiry, lest our premises should be drawn out into consequences which they do not warrant, or our credit be aspersed with doubts or suspicions that we do not intend to deserve.

The pith of Dr. Wigan's speculations will be found comprised between pp. 24—48 of his work, entitled, "*Duality of the Mind.*" Before proceeding any further, we must, on the very threshold, protest against the term,

* It is possible that the decapitated head may think, and be, for a little while at least, conscious, after the guillotine has fallen, and severed it from the trunk. In this breathless state what thoughts must come! although perhaps not more awfully supernatural than those which flit through the brain *in articulo mortis.* This may be one reason why we do not always understand the meaning of death-bed words, as it is impossible for the living to be placed in relation (*en rapport*) with the moribund, when time shall be no more.

The decapitated head may, for a few brief seconds of time, have the power of willing, although not of executing its will. The question of the power of the human will is a very recondite one, and is more likely to be at last satisfactorily cleared up by physiology than by theology and metaphysics. "*What you will, that you are,*" was an aphorism of St. Augustine's, not unlike the words of Virgil, "*possunt quia posse videntur,*" so cleverly translated, "*they only conquer who believe they can.*" The vulgar adages—"*Where there's a will, there's a way,*" "*You can, if you will,*" "*A wilful man maun have his way,*" are each of them expressive of the absolute power of the human will. There can be no doubt that the will is exerted by the silent influence of the eye; and hence the well-known phrases, "*favourable regards,*" "*within eye-shot,*" "*a suspicious glance,*" "*I could tell, if I could catch his eye,*" "*a love-look,*" "*good looks,*" and "*bad looks,*" the malign influence of the "*evil eye,*" and the delightful presence of the "*happy eye.*" A fixed intentional gaze upon a person whose back is towards us, will eventually compel him to turn his head round, *to see* who is *looking at him.* Any one with a strong will may try this experiment for himself. If we fix our eyes upon a dog for some time, the animal will bark, or a cat will slink out of our sight. There is an expression of "*staring him out.*" It is said that savage animals may be overawed by the human eye. The will of great personages, such as Napoleon, whose name struck terror into every cabinet in Europe, is not due to the actual presence of the party exerting his will. Its potency extends far beyond the narrow limits of corporeal presence or personality. It is the act of willing, *per se*, that is operative, exclusive of space. Thinking intently on another afar off, causes emotion of mind in the person thought of: hence people say, "*Somebody is talking or thinking of me;*" and the approach of some one with whom we are acquainted or concerned, is preceded by the sensation or thought of that person, according to the popular sayings, "*talk of the d—l,*" &c., "*coming events cast their shadows before them,*" "*quand on parle du loup, on en voit la queue.*" Hence, likewise, the doctrine or superstition of presentiments, omens, presages, forecastings, second sight, &c. But the strangest exertion of the human will is that of fortune-telling, unanimously interdicted by common sense, the Christian religion, and legal enactments. Soothsayers, or seers, have, however, always existed, from the witch of Endor to the unhappy wretches who were burnt to death or drowned for witchcraft in the middle ages, and from thence down to the clairvoyants of modern mesmerism. Their existence, and the truth, real or imaginary, of their prognostics, are beyond question. There are some striking predictions of this kind upon record; and the universal consent with which they have been received in all ages, only proves their agreement with the experience or the feelings of mankind. It is unphilosophical to deny an opinion universally entertained, however little it may accord with our private notions of the subject; neither does occasional fraud or fallacy invalidate the main evidence in its favour. Some very interesting predictions of this sort are quoted from Forbes's "*Oriental Memoirs,*" in the *Zoist* for July, 1847. Facts are stubborn things, and popular language contains the real philosophy of a nation.

*duality of mind,* which we have just shown to be impossible by reason of the mind itself being an entire whole, incapable of subtraction, division, multiplication, or addition. Dr. Holland has defined the inquiry more correctly by the term of "*the brain a double organ.*"* Dr. Wigan commences by saying, that it is—

"Entirely unphilosophical, and tending to important errors, to speak of the cerebrum as one organ. The two hemispheres of the brain are really and in fact two distinct and entire organs, and each respectively as complete, (indeed, more complete,) and as fully perfect in all its parts, for the purposes it is intended to perform, as are the two eyes. That some of the powers and functions may be combined in the medulla oblongata, or in the protuberances which occupy the cavities at the base of the bony cranium, is no greater objection to the absolute completeness and individuality of each hemisphere of the brain, or evidence of their forming but one organ, than the fact of our seeing only one object with two eyes proves that the two eyes are not distinct, complete, and separate organs, each capable of acting alone when its fellow is injured or destroyed. I shall in future speak (says Dr. Wigan) of the two cerebra, instead of the two hemispheres."—Pp. 24, 25.

Then follow twenty propositions, which, for the sake of brevity and perspicuity, we have reduced to four chief heads, as follow:—

1. That each cerebrum is a distinct brain *per se.*

2. That each has a separate and distinct power of thinking, synchronously and in concord with its fellow, or not, as may be.

3. That one cerebrum is alway superior to the other, even in health.

4. That one may be disordered or diseased, to a certain extent, without at first implicating or deranging its fellow.

These four propositions constitute the sum and substance of the author's very ingenious and interesting speculations. We own we are not quite prepared to go the full length with him on every point that he proposes to our assent. The inquiry is a novel one, and, as yet, still *sub judice.* Thus, it is not clear that we ought even so much as to admit, as a just proposition, that "in the insane it is almost always possible to trace the intermixture of two synchronous trains of thoughts" (p. 27) as necessarily indicative, or a consequence of, the two cerebra or hemispheres acting apart the one from the other. But, at the same time, we readily agree, that "while the brain is considered as the structure of one organ only, there is not much hope of any improvement in" this branch of "our physiology," (p. 30.) For it seems conclusive "that the provision of two distinct and perfect brains" is as requisite "as the provision of two ears and two eyes. We carry on only one train of thought in both brains, each thinking at the same time. All this, however, is contingent, not only on the perfect health of the organs, but on their due exercise and cultivation. In disorder and disease, brain, eye, and ear convey separate, distinct, conflicting ideas—one or both necessarily erroneous," (p. 35.) We might take some exception against the logical accuracy of this passage; for the eye receives and conveys an *image,* and the ear a *sound;* but the idea† conceived in con-

* Notes and Reflections, chap. xii.

† *Idea* is, in common language, used indiscriminately for *notion*, which is a combination, or result, of two or more ideas. Ιδεα is the picture or given object impinged upon the mind—*imaged in, imagined;* and is the οψις or ειδος, spectaculum, vision, scene, of Aristotle in his Poetics. In the Greek New Testament, Matt. xxviii. 3, Ην δε η ιδεα αυτου ως αστραπη is rendered, "*his countenance was like lightning,*" in which the *idea* is lost or obscured by the English translation. See also Longinus de Sublimitate, &c., sect. xv., de Visionibus.

sequence of the sound or image is formed within the mind. In other respects, we entirely agree with the statement. "Ratiocination is so essential to the well-being of the individual, that the possession of two organs for this purpose, each capable of carrying on the functions when its fellow is impaired or annihilated, seems only one more of the superabundant examples of design and contrivance in the structure of man, as a provision against accident or disease." (p. 35.) For our part, we believe that the duality of the brain has a still higher office than that of mere doubleness, as we shall attempt to show further on.

The author then proceeds to illustrate his propositions by a series of cases, gathered partly from his own experience and partly from the experience of others; all of them tending to establish the truth he wishes to have proved—namely, that one hemisphere of the brain, or rather, that one-half brain, is alone sufficient for all the purposes of will, memory, and understanding. There is not one of these very interesting and frequently touching cases that we would wish to have untold; for, indeed, it seems the happy gift of this writer to tell a story uncommonly well; all the chief points being brought out strongly into view, and a lively picture of the scene left deeply impressed on the memory. His feelings are always right and his perceptions clear. If we have any objection to make, it is against a certain air of jocularity, (as at page 455,) which is seldom graceful in a serious narrative. Gibbon, Bayle, and Byron, have each of them blemished their unrivalled productions by indulging in ill-timed pleasantries; and, exalted as their respective stations in life undoubtedly were, such puerility betrays a want of breeding or knowledge of the world. The first case that attracted Dr. Wigan's notice happened in early life. It was that of a boy who lost a large portion of his brain with nearly the whole of the parietal bone from an accident, and yet perfectly recovered the use of his senses for a certain length of time afterwards. But the most remarkable case is one related by that amiable physician, Dr. Conolly, which we must quote verbatim:—"A gentleman, from applying St. John Long's embrocation to the cheek for some ailment in the part, established so serious a disease that it spread through the orbit into the cerebrum, and by very slow degrees destroyed his life. He was a man of family and independence, and lodged with Mr. Gill, the tailor, now of Holles-street, Cavendish-square. On examining the skull, one brain was entirely destroyed—gone, annihilated—and in its place (in the narrator's emphatic language) 'a yawning chasm!' All his mental faculties were apparently quite perfect. Mrs. Gill (the only person he would permit to attend upon him) declares, that his mind was clear and undisturbed to within a few hours of his death. He had a perfect idea of his own awful situation, and Mrs. Gill having been gradually accustomed to the sight of the horror, was alone allowed to come near him; he would not even permit his own sister or other relatives to witness his frightful condition. This single case is conclusive." (p. 41).

There is a brief solemnity about this simple narrative that partakes of the sublime, and is one of those sad scenes in private life from which the medical man seldom draws aside the curtain except for a benevolent purpose, which, while it serves to fix a doubtful point in pathology or practice, cannot fail in forcing consideration on the careless, and seriousness on the light. We grant that this case is conclusive, for this irrefragable reason, that one arm and one eye are not parallel with one brain; for one half of the brain can perform all the functions of a double or entire brain perfectly to the last; but one eye does not see so well as two eyes, nor can one arm alone do all that is usually done by the two arms together: it is conclusive, on the contrary, that half a brain is capable of all the mental functions complete.

Gall's cases are too well known now to require repetition here.* There are some interesting cases from that most picturesque of all writers, Cruveilhier, whose authority is in this instance of so much the greater weight, as his reports bear directly on the point in question, without his having been influenced, it would seem, by any preconceived notions on the subject. If the following remark be valid, it is one of the highest practical importance:—"*In all cases of extensive or even slight disease of the cerebrum, there is observed the inability to exercise continuous study, or to learn by heart.*" (p. 47.) Leaving this very important remark to speak for itself, we will proceed to take a review of the comparative anatomy of the brain as a double organ.

The existence of animals the lowest in the scale of animated nature, seems to be for no other purpose than that of mere sustenance and multiplication by offsets or germs. In them there appears (as in the *hydra vividis*) no trace of a nervous system, at least none that in the present state of our knowledge we can properly define as such, unless the fine cord round the mouth be a nervous filament; nor can we ascertain anything like intelligence. They are scarcely raised above the vegetables. The *ascaris*, whose life is as limited as any living creature's can be well imagined, presents two white cords. The *asterias*, or *star-fish*, has a circle of ganglia or brains, from which radiate distinct nerves. This may be only the sympathetic, whose existence may be independent of a brain. Many of the molluscous creatures (*tunicata*), but little raised above the sponges, and fixed to a rock all their lives long, have nothing like a brain, particularly not a dual one. The oyster is the first to exhibit a double brain, only the two brains are separate, without a commissural connexion, unless the œsophageal arch can be regarded as such. It is singular that with this approximation to a twofold brain, Garner should have pretended to show distinct organs of vision on its mantilla or beard. But no sooner are feet produced, than an additional portion of brain is bestowed in correspondence with these members—the *pedal ganglion;* which marks a kind of epoch in the history of the nerves; for where there are feet, there is also progression; and the act of progression implies an object of desire to be sought for and obtained only by judgment, comparison, and volition. A more organized brain is therefore requisite. Thus, the common slug has two cephalic ganglia, evidently united by a small, though distinct commissure. The brain becomes a double organ. Some exceptions may be made to this order as an established law; for the *myriapoda*, or *centipedes*, which are higher than the slug, have several brains—one to each leg; while the crab, still higher than these, has only a single brain, but then its large pedal

* Gall, sur les Functions du Cerveau, tome ii. pp. 246—269. Paris, 1825.

ganglion is almost a second one. The supra-œsophageal brain of the *pearly nautilus* is duplicated, and in the *cuttle-fish* this duality is still more distinct. As soon, however, as the sensorium becomes a much more valuable organ, we arrive at those creatures which enjoy a brain in a brain-case, (*myelencephala.*) These animals cannot live without a skull, box, or casket, on purpose for holding their seat of intelligence. They no longer subsist like mere vegetation, but exist by intellectual pursuits, as in fishes, reptiles, birds, &c. The brain is now invariably a double organ, more or less perfect, and generally united by commissural bands —whiting, cod, eel, skate, &c. In the frog this is very evident. But, nevertheless, the commissures are as yet lost or confounded in the close proximity of the hemispheres. In birds, though the two hemispheres are more manifest, yet the *corpus callosum* is wanting, as in marsupials. The organ of comparison is defective, and the judgment low. In the beaver, however, with its constructive propensities, intelligent conduct, and provident habits, not only is the brain decidedly double, but the corpus callosum or organ of comparison is proportionably large. Convolutions are likewise visible. As we go on ascending in the scale of organic intelligence, the hemispheres become more distinctly double, the commissures larger, and the furrows deeper. In the elephant, so renowned of old for its understanding, and in the porpoise, so remarkable for its sagacious tenderness in nursing its young, all these characteristics are particularly visible.

Enough is all this to show, that comparative anatomy attests a truth which we were first led to assent to on the slender grounds of induction and analogy. In man, with his large brain and exalted intellect, the furrows are decided, the commissures bold and strong, and the brain a double organ,—all its intricate foldings are, as a sculptor would say, deeply chiselled and finely finished off.* "It is further to be noticed, as an anatomical fact," says Dr. Holland, in a note quoted from Meckel, "that in the brain and spinal marrow, the external parts on the two sides are less exactly symmetrical than those within; the surface of the brain showing this perhaps more distinctly than any other part."† Every one may find an opportunity of observing a difference in the relative size of the two sides of the head of some of his acquaintances; nor does this disparity or inequality seem to be detrimental to the intellectual development, but, on the contrary, rather favourable to it; for persons of distinguished talents have had their heads larger on one side than the other,—as Cicero and Bichât, for instance.‡ Indeed, some go so far as to fancy, that whenever this inequality exists, the understanding is much better than in those whose heads are more exactly symmetrical.

We have now gone through *all* that is absolutely *known* concerning the brain as a double organ; the rest is only conjecture, or if it be anything more substantial than that, it will belong to that lucky class of guesses which sometimes outstrip themselves by anticipating the truth.

The greater part of Dr. Wigan's book is taken up with histories of

* Müller's Elements of Physiology, by Baly, 2nd edit. 1840, pp. 813*—824*. The Human Brain, by S. Solly, Esq., 2nd edit. 1847, pp. 30—96.

† Notes and Reflections, chap. xii.

‡ Gall sur les Fonctions du Cerveau, tome i. p. 194, tome ii. pp. 319, 320.

cases, by which he endeavours to illustrate or verify his remarks. Perhaps there is something of a rambling spirit about him, but perhaps likewise the subject would not permit of his treating it more methodically. He has chosen an elevated spot, unfrequented, or, at least, unexplored; and as there was no direct pathway to guide him to the point he had in view, he was driven to wander hither and thither in the laudable hopes of finding one. We are much obliged to him for his narrative, as well as for the ingenuousness with which it is told.

But as travellers see strange sights, so Dr. Wigan has seen some wonders that appear passing strange to us who have been sitting all the while at home at our ease. We confess we do not understand how one brain could believe Christianity while the other did not, (pp. 189—210;) neither could we ever persuade ourselves that we knew beforehand what God intends, (p. 412,) for who knows the mind of the Almighty? Nor do we perceive that the pathology of hydrophobia receives any clearer explanation than ordinary upon the gratuitous hypothesis of one brain being more infected with the disease than the other, (p. 330;) while we hesitate very seriously at admitting the assertion, "that the distinction between mind and instinct consists in the parity or disparity of the two cerebra," (p. 347.) Likewise the phrase, "*moral madness*," has always appeared to us a very equivocal one. Every madman is morally insane; because, if his morals were sound, he would not be insane. "*Intellectual madness*" cannot be accepted as a distinct term; for erroneous intelligence arises either from partial or defective information, upon which it is morally improper to act, or else it springs from erroneous moral motives, *i. e.*, moral madness. Neither can we allow of the phrase "*physical madness*," which necessarily involves in its consequences errors both of a moral and an intellectual nature. It is necessary to define our terms with accuracy, especially upon such a subject as madness. It is not our intention to make any remarks upon madness in the present article, which is particularly devoted to the consideration of the brain as a double organ of mind; but mania is a very inviting subject, more especially that of a religious cast, which afflicts so large a class of the community in this country.* Men of the world waive the subject of religion as the topic of weak minds; but the medical man and the philanthropist know full well that the vital truths of revelation form too intimate a part of our nature to allow of their being discarded with indifference, or coldly treated with levity and disrespect. This life is better than our estate,

* That religious insanity is much less common in Romanists than Protestants, and especially Protestant dissenters, cannot be doubted by any one whose sphere of observation has enabled him to form any opinion on the matter. Dr. Hallaran (Pract. Observ. on Insanity, Cork, 1818, p. 32) states, that in the Lunatic Asylum at Cork, in which the admission of Romanists are about ten to one of Protestants, no instance has occurred, within his recollection, of mental derangement of the former from religious enthusiasm; but that several dissenters from the established church have been so afflicted. The reason of this difference is obvious. The ministers (*priests*) of the Romish (*Roman-catholic*) church do not allow the minds of their flocks to distrust points of doctrine and discipline, or to fall into those doubts which distract the minds of those who are either wavering in their opinions, or entertain entire liberty of conscience. (Dr. Copland's Dic. Pract. Med., article Insanity, § 290. See also the Appendix of Dr. Wigan's work on Duality of Mind, an article on Religious Insanity, in which Dr. Wigan agrees with Dr. Copland in this view of the matter.)

and the next life is better than this—this is the burning truth that scorches to death the brain of many an earnest, ardent soul, account for it as we may. To mock at the heaviest of human afflictions, says the sage Imlac, is neither charitable nor wise. For of the uncertainties of our present state, the most dreadful and alarming is the uncertain continuance of our reason.

A curious instance of literary duality was pointed out by the late Bishop Jebb, in the parallelism of Scripture. All the higher sentiments are, when strongly expressed, dual or double, and frequently, in their most energetic exhibitions, triple, until, after running up into a climax of passion, they descend according to a true chromatic scale, and end in a cadence note, which is so essential to the emphasis that it fails in producing the proper effect if the cadence be either false in pitch or inadvertently omitted in the rhythm. And not only is this falling-note thus absolutely necessary to the right expression, but it must likewise conclude by repeating the first movement or emotion (a real *da capo*) with which the burst of feeling was first given vent to. The best example that can be adduced in illustration of this remark will be found in the parable of the "Prodigal Son," the whole of which may be worked out into a series of double or triple parallelisms, till it ends in the recurring melody of "*was dead, and is alive again; was lost, and is found;*" which brings back the mind to the first idea of the prodigal leaving his home, with a pathos of the happiest and most touching kind.* Now, this duality, so exquisitely beautiful in poetry of an epic character, has most likely its exact counterpart in the structure of the material organ of the mind by which it was conceived—the brain.

It is singular that the brain, as a double organ, has not received closer attention than it has hitherto done. We candidly own, that, with all its faults, "which, light as straws, upon its surface flow," we have derived more information from Dr. Wigan's speculations than from any other work that we have opened on the subject. The reason of the duality of the brain not being more particularly examined into, is, in all probability, owing to almost all the other organs of the body being double, at least, those most commonly exposed to view—*e. g.*, the eyes, the nostrils, the hands, &c. But of the viscera occupying the three great splanchnic cavities—the abdomen, the thorax, and the calvarium—the brain is the only one that is really twofold. For the lungs are not justly so, nor is the heart, nor the stomach and bowels, but only the kidneys. There must be some reason for this. That this duplication is in most instances a natural precaution, that in case of one being injured, a second should remain for carrying on the functions proper to

* Many other instances of a similar parallelism in literature or art might be adduced, especially in architecture, in which the eye is never satisfied, unless the grand outlines of the building are doubled or repeated on either side. This is one reason why the Parthenon, which is a parallelogram divided into two symmetrical halves by the obtuse angle of the pediment, produces the most delightful effect, whereas the triple temple of the Erectheum, Minerva Polias, and Pandrosium, exquisitely detailed as it is throughout, fails in producing the same happy impression, because its parts or projections are irregular and unaccountable. The servile manner in which this latter edifice has been copied in the present age, is only a proof of the inherent bad taste of modern architects. *Decipit exemplar vitiis imitabile!*

the part, or necessary to life, is not a sufficient reason to account for the duality of the brain, however well it may serve to account for the kidneys, or any other organ; because the brain is more particularly an entity in its twofold capacity than the kidneys can be said to be, or the mammæ, or the hands, &c.—these organs being more like *duplicates* than *duals*—whereas the brain is, in fact, *but one organ*, not duplicate, but *dual* in itself. The duality of the brain has more the character of the treble and bass in musical composition; or the corollary, which is a perfected reflection of the theorem in mathematics; or the countersign of legal documents in attestation of the validity of the sign-manual. These are dualities, not repetitions or duplicates. Reasoning *à priori*, we should be disposed to predicate that the brain ought, as the ultimate organ of sensation, and the chief instrument of thought and will, to be twofold; because two things are necessary to produce a third; and the third or product is, when generated by the second or factor, the result of the first or prime number; for if the brain were a unit, instead of a dual, it must remain an inert unit, inasmuch as unity cannot be reckoned as a factor. Its duality is involved in the very terms of its definition—the organ of will, memory, and understanding. Thus, our deeds attest the validity of our judgment, which is the result of a comparison of things within the mind by a twofold instrument of thought—the brain.

The grand question of materialism that agitates sensitive minds so much is an immaterial one; for the immaterial principle must be expressed by the material agent, just as the material agent must execute the act agreed upon by both; yet no one will affirm, we suppose, that the act is the agent, any more than the agent is the principal, although in action they are all three of them one and the same. This runs up into a question of duality of the highest description, terminating in a trinity, or the perfect number, without which there can be no such thing as arithmetical progression or animated existences in the world.

We perceive, says Gall,* that no faculty of the mind can manifest itself without material agency—that every faculty, even those called the spiritual, can act only through the means of matter, and that their action cannot be perceived except by material organs. St. Thomas Aquinas,† in reply to those who confused the power with the agent, says:—Albeit spirit is not corporeal, yet the operations of the spirit, such as memory, thought, imagination, &c., cannot be performed but by corporeal organs. On this account it happens, that when an organ cannot, through some accidental derangement of its parts, continue to act, the operations of the spirit are likewise deranged together with it, as in delirium, asphyxia, &c. And thus also, the healthy organization of the human frame ensures the distinguished faculties of a healthy intellect as their necessary consequence or result—the *mens sana in corpore sano*. Plato, in his Timæus,‡ had arrived at a somewhat similar conclusion, when he calls the universe the paradigm of the eternal being, as our bodies are the paradigm of our souls; the lustre of the soul being in exact ratio to the more or less perfect execution of the paradigm, or, as St. Paul calls it,

* Sur les Fonctions du Cerveau, tome i. p. 231.

† Contra Gentiles, c. 84, n. 9, quoted by Gall, *ut supra*.

‡ Taylor's Plato, vol. ii. 4to, London, 1804, p. 490.

the "earthly house of our tabernacle"—*terrestris domus nostra hujus habitationis.* (2 Cor. v. 3.)

Age after age passes away, and generation after generation sinks into the grave, while science goes on slowly evolving truth—truth that lives and burns higher and brighter and fairer as all things else decay. The school of Athens, at its happiest period, shone with pale effulgence on the morning of the world. Its master, Socrates, hesitated at pronouncing a conclusion, (*causa causarum,*) which constitutes the fundamental dogma of every sect in science or religion at the present day, and at last died for declaring an article of faith, (*credo in unum Deum,*) which every boy would now be flogged for not believing. The progress of knowledge is slow. Centuries are consumed in teaching a solitary truth, (*iterum venturus est cum gloria judicare vivos et mortuos,*)—in unlearning or repenting of the mistakes of inexperience, (*non enim sciunt quid faciunt,*)—or in uprooting the still deeper errors of prejudice and birth, (*quod ergo ignorantes colitis, hoc ego annuntio vobis.*) We die in our ignorance to-day, in order that others may live on instead more wisely than ourselves to-morrow. Were nations or individuals to be immortal on earth, vice would be the standing order of the day, and ignorance as certain as the night. As time goes forward, all truth tends to coincide with the divine word: and, if we do not always discover the successive coincidences so repeatedly occurring at present, it is because we are not yet sufficiently far advanced to discern all the connecting media. But as repeated coincidences imply a law of connexion, so it is not impossible that the connexion between profane learning and revealed truth may at length prove itself to be conclusive and unique. For, according to a just train of reasoning, it is quite impossible that the splendid fragments of pagan verity, doubtless of primeval origin, as well as the luminous agenda of modern science, doubtless, likewise, of more than earthly mould, should not, according to the well-known order of Providence, be drawn together and circulated around the great centre of truth itself, by the divine force of their own intellectual affinity.

